# Bioinformatic Analysis Identified Potentially Prognostic Long Noncoding RNAs and MicroRNAs for Gastric Cancer

**DOI:** 10.1155/2021/6683136

**Published:** 2021-12-10

**Authors:** Jiao Guo, Yongda Liu, Ping Zhao

**Affiliations:** Department of Anesthesiology, Shengjing Hospital of China Medical University, No. 36, Sanhao Street, Heping District, Shenyang, 110004 Liaoning Province, China

## Abstract

Gastric cancer (GC) is the fifth most common malignant tumor in the world. The present study was performed to discover the potential diagnostic and therapeutic long noncoding RNAs (lncRNAs) and microRNAs (miRNAs) of GC. Data used in this study to identify differentially expressed lncRNAs (DElncRNAs) and miRNAs (DEmiRNAs) were obtained from 187 GC tissues and 32 adjacent nontumor tissues. The total clinical data on GC included 187 cases. The above data were from the TCGA database. RStudio/Bioconductor software was used to conduct univariate analysis, the least absolute shrinkage and selection operator (LASSO) Cox, and multivariate Cox proportional risk regression for the DElncRNAs and DEmiRNAs. Clinical information was analyzed through univariate and multivariate Cox analysis. Results: five lncRNAs (AC007785.3, AC079385.3, LINC00392, LINC01729, and U95743.1) and two miRNAs (hsa-miR-3174, hsa-miR-605) were proven to be independent prognostic indicators of GC. Results of the Kaplan-Meier survival analysis showed that AC007785.3, AC079385.3, LINC01729, miR-3174, and miR-605 were significantly correlated with OS of GC. The target genes of AC079385.3, miR-3174, and miR-605 were obtained and clustered mainly on MAPK and cGMP-PKG signaling pathways. The clinical data showed that age and clinicopathologic stage were correlated with the prognosis of GC. Furthermore, AC007785.3 was associated with metastasis of GC, and miR-3174 was associated with the primary tumor condition of GC. We identified three lncRNAs (AC007785.3, AC079385.3, LINC01729), two miRNAs (miR-3174, miR-605), and clinical factors related to the pathogenesis and prognosis of GC. Our predicted results provide a possible entry point for the study of prognostic markers for GC.

## 1. Introduction

Gastric cancer (GC (GC: gastric cancer)) is the fifth most common malignant tumor globally (1). GC was estimated to account for 7.2% of all cancers in men and 4.1% in women in 2018. The prognosis for GC patients is poor due to the lack of effective early detection and recurrence biomarkers. Carcinoembryonic antigen, carbohydrate antigen (CA) 199, and CA724 are not ideal markers due to their relatively low sensitivity and specificity; although, they are the most commonly used (2). Therefore, more specific and sensitive novel markers for GC to establish screening strategies and individualized therapies for patients still need to be urgently identified.

Long noncoding RNAs (lncRNAs (lncRNAs: long noncoding RNAs)) are a class of RNAs that are longer than 200 nucleotides but do not encode proteins. MicroRNAs (miRNAs (microRNAs; lncRNAs)) are a class of endogenous small 19–25 nucleotide noncoding RNAs that combine with the 3′-UTR of target genes, leading to inhibition of translation or degradation of the target genes (3, 4). Since the discovery of lncRNAs and miRNAs, studies have emphasized the importance of their dysregulated expression in metastasis, development, and prognosis of various cancers, including GC (5–14). Several researchers have worked on discovering potential lncRNAs and miRNAs associated with the recurrence and survival time of GC patients, respectively, with data collected from individuals or from the public databases. H19, HOTAIR, CCAT1, GHET1, CDKN2B LSINCT-5, CUDR, LINC00152, and MALAT1 are considered to have oncogenic roles in GC, whereas GAS5, MEG3, BM742401, and FER1L4 might act as tumor suppressors. miR-1, miR-17-5p, miR-16, miR18a, miR-20a, miR-21, miR-25, miR-27a, miR-34a, miR-92a, miR-100, miR-103, miR-106a, miR-106b, miR-107, miR-146a, miR-148a, miR-192, miR-194, miR-196a, miR-199a-3p, miR-200c, miR-210, miR-221, miR-223, miR-376c, miR-378, miR-423-5p, miR-421, miR-451, miR-486-5p, miR-744, and miR-93 are upregulated in the circulation of GC patients. However, miR-195-5p, let-7a, miR-218, miR-375, and miR-122 are downregulated in the circulation (15).

The use of circulating molecular profiles as potential biomarkers for the diagnosis and prognosis of GC is still not recognized. On this basis, to exclude effects of chemoradiotherapy and individual differences in the published studies and to avoid the intervention of blood samples, we identified differentially expressed lncRNAs and miRNAs through bioinformatics analysis. In this study, we used data from the same patients' tissues from the cancer genome atlas (TCGA (TCGA: The Cancer Genome Atlas)) data portal.

## 2. Materials and Methods

### 2.1. Data Preparation

The RNA information and clinical data used in this study were from the cancer genome atlas (TCGA) data portal (https://tcga-data.nci.nih.gov/tcga/). Therefore, approval from an ethics committee was not required. The inclusion criteria were set as follows: (1) samples with common and completed lncRNA, miRNA, and clinical data. (2) Patients had not received preoperative chemoradiotherapy. As a consequence, the data used to identify the differential expression of lncRNAs, miRNAs, and mRNAs in gastric cancer were obtained from 187 GC tissues and 32 adjacent nontumor tissues. The total clinical data on gastric cancer was obtained from the corresponding 187 patients.

### 2.2. Expression Profiling and Analysis of Differentially Expressed lncRNAs (DElncRNAs (DElncRNAs: Differentially Expressed lncRNAs)), Differentially Expressed miRNAs (DEmiRNAs (DEmiRNAs: Differentially Expressed miRNAs)), and Differentially Expressed mRNAs (DEmRNAs)

Firstly, the RNA sequences were downloaded from the TCGA database, and information for each sample was added to a matrix to extract the lncRNA, miRNA, and mRNA expression data through Perl. Then, the *R*/Bioconductor *edgeR* package was used to screen DElncRNAs, DEmiRNAs, and DEmRNAs with differential expression analysis. A log_2_ fold change (FC) > 1.0 and a false discovery rate (FDR (FDR : false discovery rate)) < 0.05 were set as the selection criteria.

### 2.3. Statistical Analysis of DElncRNAs and DEmiRNAs

Univariate Cox regression analysis was first used to screen the DElncRNAs and DEmiRNAs associated with the prognosis of GC. Then, the least absolute shrinkage and selection operator (LASSO) regression was performed to reduce the discreteness. A multivariate Cox proportional risk regression model was used to independently identify prognostic lncRNAs and miRNAs. Concordance index (C-index) and the receiver operating characteristic (ROC (ROC: receiver operating characteristic)) curves were taken to evaluate the model. GC patients in each dataset were divided into a high-risk group and a low-risk group according to the median cut-off points of risk. The *survminer* packages in the *R* software were used to generate forest charts. The Kaplan-Meier survival curves were plotted based on the different risk scores and expression levels of the screened lncRNAs and miRNAs. The above analysis was carried out using the RStudio/Bioconductor. A *P* value <0.05 was defined as a significant difference.

### 2.4. Statistical Analysis of Clinical Data

The clinical samples were first divided into two halves using the *caret* package. Using the RStudio/Bioconductor method, single- and multifactor Cox regression was performed in the training group using the *survival* package. The *rms* and *survival* packages were then used to generate nomogram and calibration graphs. The ROC curve was used to predict the accuracy of the model. The clinical data obtained from the training group were verified by calculating the C-index of the clinical data onto the test group. The Kaplan-Meier survival curve was used to predict the survival difference, and the effect of each clinical factor on the OS (OS: overall survival) of patients was evaluated. *P* < 0.05 was defined as a significant difference.

### 2.5. Enrichment Analysis of Target Genes

Detailed information about the screened lncRNAs was further searched for (https://genome.ucsc.edu). The target miRNAs of the screened lncRNAs were identified with the miRcode (http://www.mircode.org) database. Possible downstream target genes of the obtained target miRNAs and the screened prognostic miRNAs, which appeared in at least two databases, were collected from the miRDB, miRTarBase, and TargetScan websites. Then, the possible lncRNA-miRNA-mRNA and miRNA-mRNA networks were drawn based on the competing endogenous RNA theory. The functions and related enrichment pathways to the possible target genes were analyzed using the DAVID tool (version 6.7, https://david.nciferf.gov/).

### 2.6. Validation of the Prognostic Performance of the Screened lncRNAs and miRNAs

The associations between the expression levels and clinical parameters of the screened lncRNAs and miRNAs were calculated with the Kolmogorov-Smirnov test (KS test). Besides, the expression levels of the lncRNAs between the GC tissues and normal tissues were verified in GSE53137 through Prism 8.0 (GraphPad, San Diego, CA, USA) using the Wilcoxon signed-rank test. miRNAs were verified in GSE94315 and GSE78091 with the same method. *P* value was two-sided, and a *P* value <0.05 was set as the cut-off criterion for statistical significance.

## 3. Results

### 3.1. Identification of DElncRNAs, DEmiRNAs, and DEmRNAs

According to the inclusion criteria, data from 187 GC tissues and 32 adjacent nontumor tissues were obtained from the TCGA database. A total of 2865 DElncRNAs were identified, of which 2223 were overexpressed and 642 were downregulated. A total of 261 DEmiRNAs were obtained, 215 of which were upregulated and 46 were downregulated. A total of 4555 DEmRNAs were found, among which 2268 were upregulated and 2287 were downregulated.

### 3.2. Prognostic Performance of DElncRNAs and DEmiRNAs

**Figure 1:**
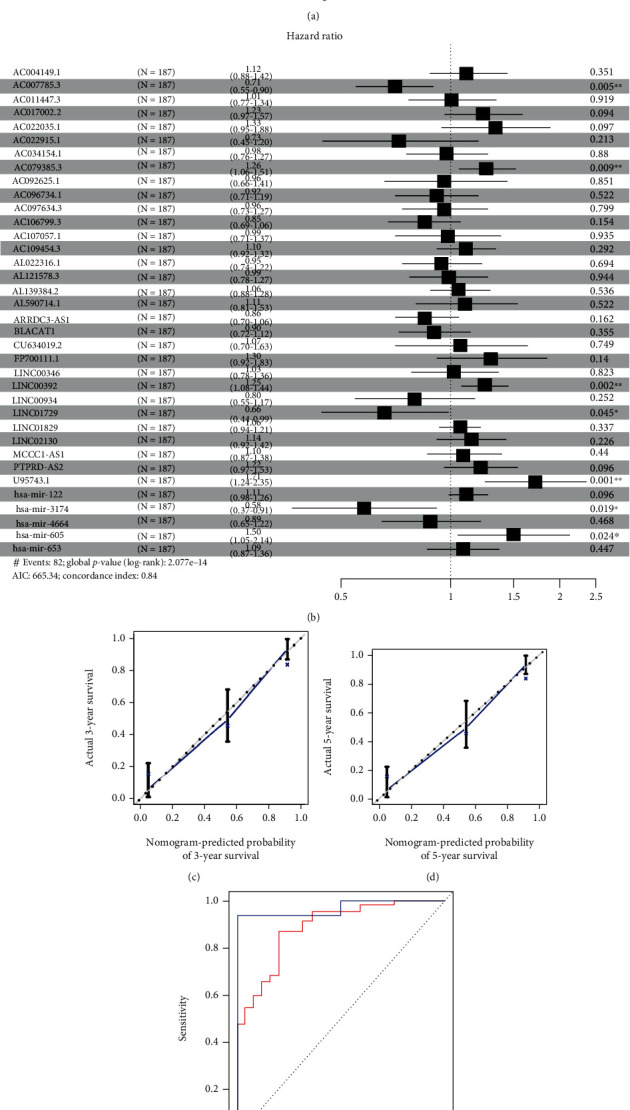
Statistical analysis results of DElncRNAs and DEmiRNAs. (a) The plot of DElncRNA and DEmiRNA regression coefficient diagram. (b) Forest map of DEGs. (c) The calibration plot for predicting patient 3-year survival. Nomogram-predicted probability of 3-year survival is plotted on the *x*-axis; actual 3-year survival is plotted on the *y*-axis. (d) The calibration plot for predicting patient 5-year survival. Nomogram-predicted probability of 5-year survival is plotted on the *x*-axis; actual 5-year survival is plotted on the *y*-axis. (e) AUC curves for 3-year and 5-year survival probability.

**Figure 2:**
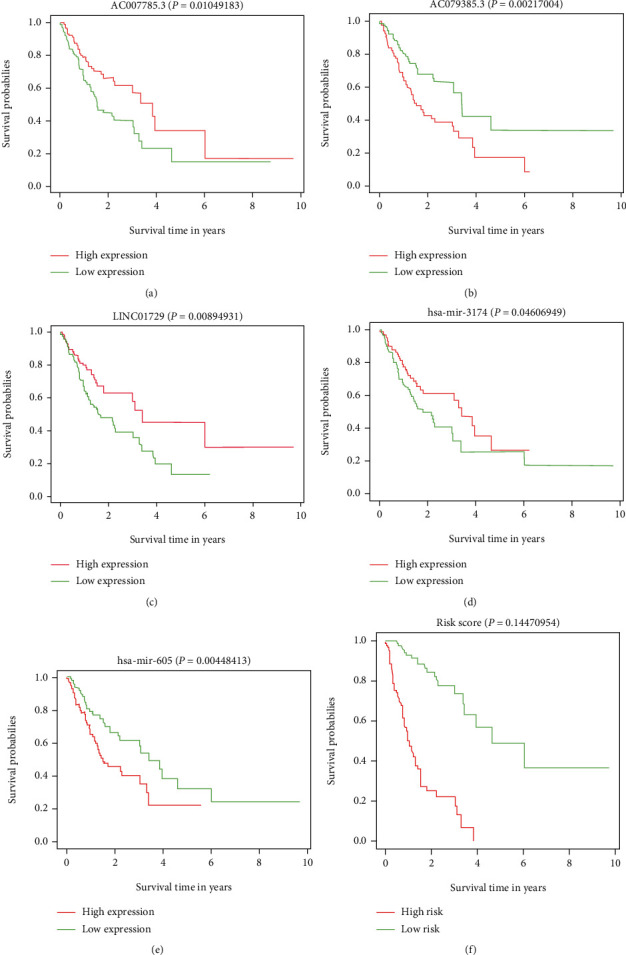
Survival curves are plotted for BRCA patients. (a) AC007785.3. (b) AC079385.3. (c) LINC01792. (d) hsa-miR-3174. (e) hsa-miR-605. (f) Survival curve for risk score.

Univariate Cox regression was performed for significant lncRNA and miRNA expression. The results showed 544 DElncRNAs and seven DEmiRNAs that were associated with OS of GC patients. Next, we used LASSO regression to further reduce the discrepant, and 35 DElncRNAs and 5 DEmiRNAs were screened. The multivariate Cox proportional risk regression model was used to verify the above results and identify independent prognostic factors. The results of the multivariate Cox proportional hazard regression analysis are shown in Figure 1(a). The C-index of the model was 0.84. We found that five DElncRNAs (AC007785.3, AC079385.3, LINC00392, LINC01792, and U95743.1) and two DEmiRNAs (hsa-miR-3174 and hsa-miR-605) were statistically meaningful (Figure 1(b)). We also used time-dependent ROC curves to assess the prognostic ability of the screened biomarkers (Figures 1(c) and 1(d)). The AUCs (AUC: area under the ROC curve) of the prognostic model were 0.89 and 0.968 for the 3-year and 5-year OS, respectively, revealing high prognostic performance (Figure 1(e)). Then, three lncRNAs (AC007785.3, AC079385.3, and LINC01792) and two miRNAs (hsa-miR-3174 and hsa-miR-605) were proven to have meaningful survival curves (Figure 2).

### 3.3. Enrichment Analysis of the Screened Biomarkers

**Figure 3:**
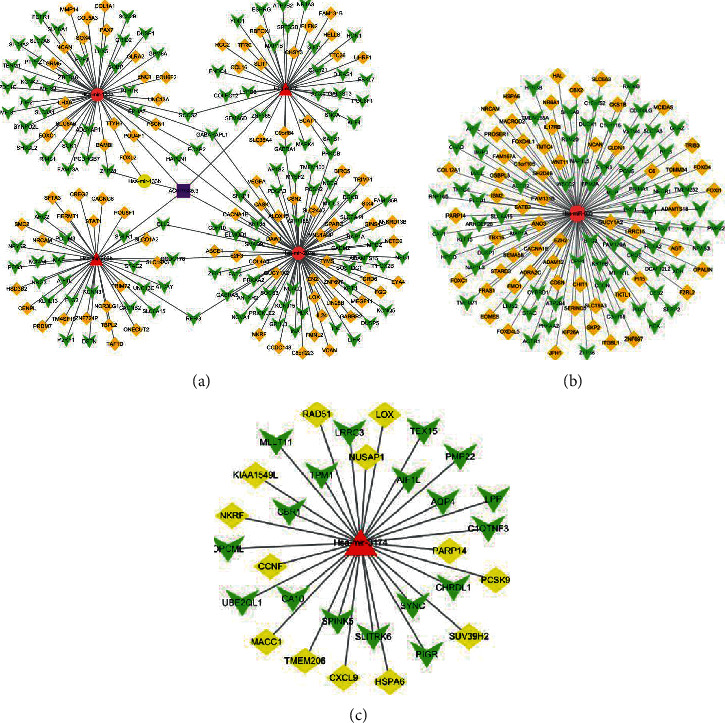
The possible lncRNA-miRNA-mRNA and miRNA-mRNA networks and hub clustering modules. (a) AC079385.3. (b) hsa-miR-605. (c) hsa-miR-3174.

Among the three screened lncRNAs, only the target miRNAs of AC079385.3 were found in the miRcode database. Then, five miRNAs (miR-7, miR-133a, miR-133b, miR-203a, and miR-203b) were obtained by taking the intersection with the 261 DEmiRNAs in our study. Finally, 212 possible target genes of the five miRNAs were explored. The lncRNA-miRNA-mRNA network is shown in Figure 3(a). At the same time, 31 and 129 possible target genes were obtained for the miRNAs. The miRNA-mRNA networks are shown in Figures 3(b) and 3(c). The functional analysis in Table 1 lists the top five results of the gene function analysis. The pathways to gene enrichment are shown in Table 2.

### 3.4. Clinical Data Mining

**Figure 4:**
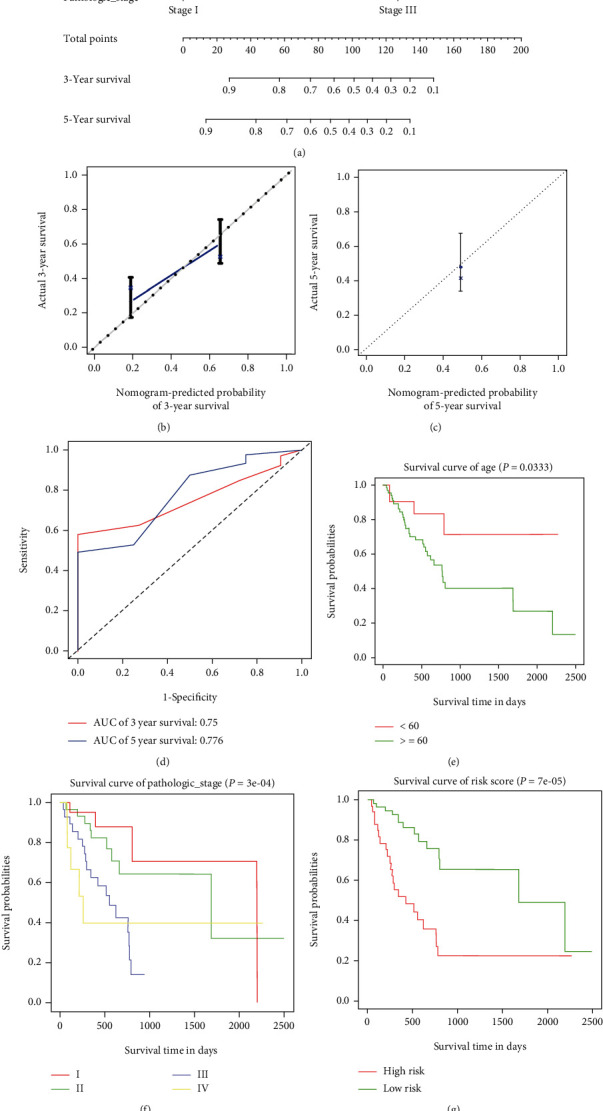
Statistical analysis results of clinical data. (a) The nomogram of clinical data for predicting proportion of patients with 3-year and 5-year survival. (b) The calibration plot for predicting patient 3-year survival. (c) The calibration plot for predicting patient 5-year survival. (d) AUC curves for 3-year and 5-year survival probability. (e) Survival curve of age. (f) Survival curve of pathologic stage. (g) Survival curve of risk score.

Clinical data on the 187 gastric cancer patients were taken for analysis, with 95 patients in the training group and 92 patients in the test group. The clinical features of the gastric cancer patients in the training group and test group are shown in Table 3. Multivariate Cox analysis was constructed after univariate Cox analysis, and the results are shown in Figure 4(a). Then, the nomogram with meaning was drawn, and the 3-year and 5-year survival calibration graph evaluation model was performed to evaluate the model as shown in Figures 4(b) and 4(c). The C-index of the multivariate model in the training group was 0.739, and AUCs were 0.75 and 0.776 for the 3-year and 5-year OS, respectively (Figure 4(d)). To fully assess the prediction ability of the model, the C-index of the test group was 0.718, revealing moderate prognostic accuracy. In addition, the survival diagram of the relevant clinical data is shown in Figures 4(e)–4(g).

### 3.5. Validation of the Prognostic Performance of the Screened lncRNAs and miRNAs

**Figure 5:**
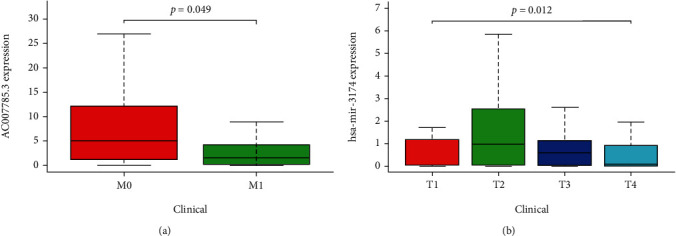
Kolmogorov-Smirnov test of lncRNA and miRNA. (a) AC007785.3. (b) miR-3174.

**Figure 6:**
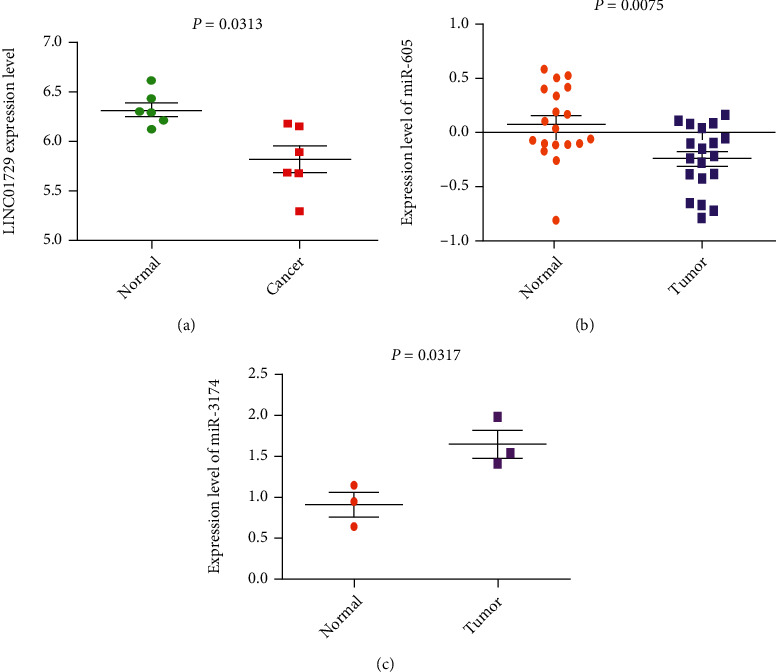
The expression of DElncRNA and DEmiRNAs between normal and cancer tissues. (a) LINC01792. (b) miR-605. (c) miR-3174.

The association between the screened lncRNAs and miRNAs and the clinical indicators were calculated with the Kolmogorov-Smirnov test. AC007785.3 was identified as being associated with metastasis of GC (*P* = 0.049), and miR-3174 was associated with the primary tumor condition of GC (*P* = 0.012) (Figures 5(a) and 5(b)). Validation of the expression of the lncRNAs and miRNAs was conducted with the Wilcoxon signed-rank test of GSE53137, GSE93145, and GSE78091, respectively. The obtained results were in accordance with our results (Figures 6(a)–6(c)).

## 4. Discussion

Gastric cancer (GC) is one of the most aggressive and lethal tumors in the world. Efforts have been made to provide new insights into the molecular mechanisms underlying GC development. Therefore, an indepth exploration of GC-related miRNAs and lncRNAs may provide clinicians with new targets for the treatment of this disease. Several integrated genetic studies have been conducted to elucidate the roles played by miRNAs and lncRNAs (16–19). In recent years, more and more reports have shown that the dysregulated expression of miRNAs and lncRNAs may be involved in tumor development, progression, metastasis (20–22), and prognosis (23–26). Some miRNAs and lncRNAs, such as miR-133a and lnc-FOXD2, have been proven to be useful biomarkers for the prognosis of GC patients (27, 28).

As described, to exclude effects of chemoradiotherapy and individual differences, we identified differentially expressed lncRNAs and miRNAs through bioinformatic analysis with the data of the same patient from the TCGA database in this study. Using univariate, LASSO, and multivariate Cox regression analyses and survival analyses based on 187 GC and 32 adjacent normal tissues, three lncRNAs (AC007785.3, AC079385.3, and LINC01729) and two miRNAs (miR-3174 and miR-605) were proven to be independent prognostic factors of GC patients.

Based on the competing endogenous RNA theory, target mRNAs of AC007785.3 (miR-3174 and miR-605) were found. Functional enrichment analysis of the target mRNAs showed that the genes clustered mainly in the plasma and play roles mainly through transcription processes. For pathway analyses, genes were associated mainly with cancer pathways and work through the calcium signaling pathway, MAPK signaling pathway, cGMP-PKG signaling pathway, and focal adhesion. Among them, the MAPK and cGMP-PKG signaling pathways were identified as being active in both the early and advanced stages of tumorigenesis, survival, promoting tumor proliferation, and metastasis in various human tumors (29). Inhibiting these pathways is considered a primary goal for clinics that provide new target therapy directions for GC.

AC007785.3, AC079385.3, and LINC01729 are not well studied, not to mention in GC. However, this offers a new direction for treatment research of GC. As we discovered, the possible target miRNAs of AC007785.3 that were upregulated were miR-7 and miR-203b, and that were downregulated were miR-133a and miR-203a. Through differential expression analysis, AC007785.3 was shown to be upregulated in GC tissues. Considering the ceRNA theory, miR-133a and miR-203a deserve more attention. Current research has found that miR-133a could inhibit the proliferation of GC cells through targeting the expression of ERBB2, FOXP3, IGF1R, and presenilin 1 and blocking autophagy-mediated glutaminolysis (30–34). miR-203a was identified as a tumor suppressor by targeting IGFIR and E2F in GC (35). Therefore, AC007785.3 might act as a ceRNA in GC.

miR-3174 and miR-605 were also identified as prognostic factors of GC. Among them, miR-3174 was highly expressed and proved to be a protective factor in our study, which was opposite to the research results of Li et al. Li et al. discovered that miR-3174 contributed to apoptosis and autophagy cell death defects in gastric cancer cells by targeting ARHGAP10 (36). This demonstrates that miR-3174 might play different roles in targeting different mRNAs. miR-605 was proven to be downregulated in GC tissues, playing carcinogenic roles in our study, which was not completely connected with current research results (37). Further research should be conducted to identify the role of miR-605 in GC.

Moreover, we also carried out an in-depth exploration of the clinical data in gastric cancer. According to the clinical data, the AUCs of the 3-year and 5-year ROC curves were 0.75 and 0.776, respectively, and the results of the disease-free survival calibration chart over 3 years were in good agreement with the results of the ideal model. Age and pathologic stage were proven to be directly connected with the prognosis of GC in our study. miR-3174 was connected with the primary tumor condition of GC.

To sum up, the biological functions of the three identified lncRNAs and miRNAs are not fully understood or elucidated in gastric cancer. However, some molecular biomarkers can predict the 5-year survival rate of patients with gastric cancer, which may become a new prognostic indicator for predicting clinical efficacy. MAPK and cGMP-PKG signaling pathways might be new target therapy directions for GC. The roles of these genes are worthy of further study because of their close association with the prognosis, especially in gastric cancer. More studies are needed in the future to verify these findings.

## 5. Limitations

This study has some limitations. Firstly, the ethnic sources of the TCGA data population are mainly limited to Caucasian and Negroid people, and inference cannot be made about other ethnicities. Secondly, in vitro and in vivo studies on these biomarkers in gastric cancer cell lines and animal models, respectively, have not been conducted. Therefore, further experimental studies are needed to strengthen the understanding of the mechanisms underlying the involvement of these markers in the prognosis of gastric cancer.

## Figures and Tables

**Table 1:** top 5 GO terms in each category enriched by target genes of GC. GO: Gene Ontology; MF: molecular function; BP: biological process; CC: cellular component.

Ontology	ID	Description	Count	*P* value	Genes
MF	GO: 0003700	Transcription factor activity, sequence-specific DNA binding	30	0.005212	POU6F1, E2F3, JDP2, POU6F2, NR6A1, SOX4, SOX6, ZEB1, ZBTB16, TBPL2, MEIS2, POU5F1, PAX7, FOXD4, MAF, FOXL2, TBX15, SMAD9, EOMES, ESRRG, ZFP28, STAT1, FOXP2, UHRF1, FOXD4L1, PBX1, FOXC1, FOXI1, KLF4, PEG3
	GO: 0043565	Sequence-specific DNA binding	25	2.82E-05	MAF, JDP2, SATB2, FOXL2, NR6A1, ESRRG, EOMES, GRHL3, EN2, SOX6, SIX4, NR4A3, FOXP2, FOXD4L1, MEIS2, POU5F1, PAX7, LHX5, POU4F1, PBX1, FOXC1, FOXI1, LHX9, FOXD4, FOXD4L5
	GO:0005509	Calcium ion binding	22	0.022085	ME1, SLC8A1, NCALD, MICU3, SNCA, DUOX1, PCDHGB7, FLG2, SPARC, MMP14, SLIT1, ATP2B2, DGKB, FAT3, SLC24A2, AIF1L, CACNA1E, VCAN, PLCD1, NCAN, CDH10, CSN2
	GO: 0042803	Protein homodimerization activity	20	0.075097	XDH, FGFR1, TBX15, CADM2, NR6A1, BIRC5, ZBTB16, NR4A3, ZNF365, STAT1, CDSN, FOXP2, TYMS, TFRC, TENM1, MAPK4, VEGFA, ADRA2C, HPGD, DPP4
	GO: 0046982	Protein heterodimerization activity	16	0.023513	CAV1, JDP2, TBX15, SOX4, BIRC5, SOX6, SMC2, MEIS1, FOXP2, AGTR1, TENM1, MAPK4, P2RY1, VEGFA, PBX1, ADRA2C
CC	GO: 0005886	Plasma membrane	112	5.6E-06	F2RL2, SLC6A1, CADM2, SLC6A3, SNCA, SLC7A8, CASK, AQP4, IL17RB, NRCAM, ATP2B2, S1PR3, AGTR1, DIRAS1, ATP2B4, MALL, KCNK7, GRID2, NEGR1, LIFR, PIM1, COLEC12, ANKRD13B, RCC2, CD300LG, PMP22, UNC13C, SH3GL2, FRAS1, ME1, NCR3LG1, FGFR1, SLC38A4, CAV1, PTH1R, KCNA4, CACNB2, RIMS1, TTYH3, FGG, SERINC5, OPALIN, P2RY1, ADRA2C, PRIMA1, HCN1, VSTM4, OSBPL3, GABRA1, LPP, GABRA5, EPM2A, SLC6A15, NPY5R, EPHA7, SEMA6D, PKP1, SFRP1, RAP1A, ANTXR2, CACNA1E, GNAZ, KCNJ15, OPCML, GLRA2, DUOX1, NBEA, GABBR2, KCNQ5, SLC24A2, GUCY1A2, ANO3, TMEM100, FAM129A, JPH1, DPP4, SGK1, CACNG8, PCDHGB7, PIGR, MMP14, PRKCB, GRM5, ALOX15, CYBRD1, ADAM12, LIMS2, TRIB3, SLC19A3, ZBTB16, GJC1, FNDC4, SLCO1A2, DGKB, GPM6A, FAT3, KRT1, PCSK9, PLCD1, HTR3B, SLC8A1, SLC8A2, KL, MAP1B, SPARC, TFRC, TENM1, CDON, KCNN3, SLC6A6, BAMBI, CDH10
	GO: 0005887	Integral component of plasma membrane	53	1.18E-06	F2RL2, KCNJ15, NRG3, SLC6A1, CADM2, SLC6A3, GLRA2, SLC7A8, AQP4, GABBR2, IL17RB, NRCAM, ATP2B2, AGTR1, S1PR3, ATP2B4, KCNK7, SLC24A2, GRID2, MPP1, LIFR, PIGR, MMP14, SLC7A14, GRM5, CLDN1, SLITRK6, FGFR1, SLC38A4, CAV1, PTH1R, KCNA4, CACNB2, SLC19A3, SLCO1A2, P2RY1, ADRA2C, HTR3B, HCN1, SLC8A1, GABRA1, SLC8A2, KL, PTPRZ1, GABRA5, SLC6A15, NPY5R, EPHA7, TFRC, SEMA6D, TENM1, CDON, SLC6A6
	GO: 0005576	Extracellular region	43	0.013279	F2RL2, FGFR1, NRG3, C6, SNCA, CXCL9, SPINK5, ISM2, CHIT1, ADCYAP1, ITGBL1, IL17RB, NRCAM, FGG, AGT, CREG2, SFTA3, COL12A1, PDGFD, LOX, FGF1, COL4A4, BMP3, HAPLN1, VSTM4, KL, SPARC, IL24, COL5A3, CCL16, SLIT1, CHRDL1, TFRC, SFRP1, TENM1, VEGFA, ANTXR2, VCAN, WNT11, COL1A1, ADAM12, NCAN, CSN2
	GO: 0005615	Extracellular space	37	0.015039	XDH, LYPD3, NRG3, ADAMTS15, SNCA, CXCL9, CHIT1, ADCYAP1, PCSK2, FGG, C1QTNF3, AGT, SOSTDC1, KRT1, COL12A1, PCSK9, PDGFD, LOX, FGF1, INA, BMP3, CES2, KL, PIGR, SPARC, IL24, CCL16, KRT35, SLIT1, ELFN2, TFRC, SFRP1, VEGFA, VCAN, WNT11, COL1A1, CSN2
	GO: 0030054	Cell junction	17	0.011212	GABRA1, CACNG8, MAP1B, GLRA2, SNCA, GABRA5, FERMT1, GABBR2, RIMS1, ATP2B2, ATCAY, SHISA9, GRID2, RAP1A, UNC13C, PRIMA1, UNC13A
BP	GO: 0006351	Transcription, DNA-templated	47	0.059463863	POU6F1, FGFR1, E2F3, JDP2, EZH2, NR6A1, TRIB3, MYEF2, CBX2, SOX6, ZBTB16, ZEB1, MACC1, HOXA2, NPAS3, POU5F1, ZNF697, PAX7, LHX5, PRKAA2, FOXD4, HELLS, NKRF, MAF, SATB1, SATB2, SMAD9, TBX15, ESRRG, EOMES, BIRC5, NR4A3, ZFP28, STAT1, PRKCB, SUV39H2, FOXP2, EYA4, UHRF1, FOXD4L1, BTG2, TENM1, PARP14, MCIDAS, ID4, PEG3, FOXD4L5
	GO: 0045944	Positive regulation of transcription from RNA polymerase II promoter	37	8.53E-05	NR6A1, ONECUT2, CASK, SOX4, SOX6, ZEB1, MEIS1, ADCYAP1, HOXA2, MEIS2, POU5F1, PAX7, P2RY1, POU4F1, FGF1, MAF, PHOX2B, FOXL2, SATB2, ESRRG, EOMES, GRHL3, KLF15, NR4A3, EN2, SIX4, STAT1, UHRF1, MCIDAS, CDON, VEGFA, FOXC1, ID4, PBX1, FOXI1, KLF4, PEG3
	GO: 0000122	Negative regulation of transcription from RNA polymerase II promoter	32	1.43E-05	FGFR1, JDP2, CAV1, EZH2, SNCA, NR6A1, TRIB3, CBX2, SOX6, ZEB1, ZBTB16, HOXA2, MEIS2, POU5F1, POU4F1, ZFP36, MAF, SATB1, FOXL2, SATB2, TBX15, EOMES, NR4A3, STAT1, FOXP2, SUV39H2, UHRF1, BTG2, VEGFA, ID4, KLF4, PEG3
	GO: 0045893	Positive regulation of transcription, DNA-templated	19	0.008447557	CKS1B, E2F3, FOXL2, EOMES, ESRRG, SOX4, SIX4, ZBTB16, STAT1, NPAS3, SFRP1, AGT, MLLT11, LHX5, WNT11, FOXC1, COL1A1, BAMBI, KLF4
	GO: 0008284	Positive regulation of cell proliferation	18	0.006934366	FGFR1, E2F3, ACER2, PTH1R, LIFR, SOX4, BIRC5, IL24, ADCYAP1, S1PR3, SFRP1, VEGFA, PBX1, ID4, PDGFD, FGF1, BAMBI, DPP4

**Table 2:** The enrichment pathways of target genes of GC.

ID	Pathway description	Count	*P* value	Genes
hsa04974	Protein digestion and absorption	8	0.002170232	COL4A4, SLC8A1, SLC8A2, SLC7A8, COL12A1, COL1A1, COL5A3, DPP4
hsa04550	Signaling pathways regulating pluripotency of stem cells	9	0.008196088	FGFR1, SMAD9, POU5F1, LIFR, LHX5, ID4, WNT11, MEIS1, KLF4
hsa04020	Calcium signaling pathway	10	0.011522204	GRM5, ATP2B2, AGTR1, SLC8A1, SLC8A2, ATP2B4, PDE1C, CACNA1E, PLCD1, PRKCB
hsa04080	Neuroactive ligand-receptor interaction	13	0.011744484	GRM5, F2RL2, S1PR3, AGTR1, GABRA1, PTH1R, GLRA2, P2RY1, GABRA5, GRID2, ADRA2C, GABBR2, NPY5R
hsa04727	GABAergic synapse	6	0.030695426	GABARAPL1, GABRA1, SLC6A1, GABRA5, GABBR2, PRKCB
hsa04924	Renin secretion	5	0.042641561	AGTR1, PDE1C, AGT, GUCY1A2, ADCYAP1
hsa04022	cGMP-PKG signaling pathway	8	0.044636113	ATP2B2, AGTR1, SLC8A1, SLC8A2, ATP2B4, MRVI1, GUCY1A2, ADRA2C
hsa05200	Pathways in cancer	14	0.061594868	COL4A4, FGFR1, CKS1B, E2F3, SKP2, BIRC5, ZBTB16, STAT1, RAD51, PRKCB, AGTR1, VEGFA, WNT11, FGF1
hsa04510	Focal adhesion	9	0.062629277	COL4A4, CAV1, VEGFA, RAP1A, PDGFD, COL1A1, COL5A3, PRKCB, CHAD
hsa04010	MAPK signaling pathway	10	0.077612358	DUSP5, FGFR1, DUSP1, CACNG8, HSPA6, RAP1A, CACNB2, CACNA1E, FGF1, PRKCB

**Table 3:** Clinical feature of gastric cancer patients in train group and test group.

Variables	Univariate analysis		Multivariate analysis	
	HR (95% CI)	*P* value	HR (95% CI)	*P* value
Gender (female/male)	0.908 (0.466-1.766)	0.777	—	—
Age (<60/≥60)	2.941 (1.038-8.333)	0.0424^∗^	6.616(1.752-24.986)	0.00532^∗^
Pathologic stage (I vs. II/III/IV)				
I vs. II	1.599 (0.492 -5.20)	0.4349	1.9412 (0.587-6.417)	0.2768
I vs. III	4.692 (1.5684-14.04)	0.0057^∗^	0.2131 (1.568-14.04)	0.00374^∗^
I vs. IV	0.2907 (0.909-13.01)	0.0687	12.833 (2.776-59.326)	0.00109^∗^

^∗^ *P* < .05.

## Data Availability

The data used to support the findings of this study are included within the article.
